# A Human Intention and Motion Prediction Framework for Applications in Human-Centric Digital Twins

**DOI:** 10.3390/biomimetics10100656

**Published:** 2025-10-01

**Authors:** Usman Asad, Azfar Khalid, Waqas Akbar Lughmani, Shummaila Rasheed, Muhammad Mahabat Khan

**Affiliations:** 1 Department of Mechanical Engineering, Capital University of Science and Technology, Islamabad 45750, Pakistan; shummaila@cust.edu.pk (S.R.); drmahabat@cust.edu.pk (M.M.K.); 2 Digital Innovation Research Group, Department of Engineering, School of Science & Technology, Nottingham Trent University, Nottingham NG11 8NS, UK; azfar.khalid@ntu.ac.uk; 3 Department of Engineering, Birmingham City University, Birmingham B4 7XG, UK; waqas.lughmani@bcu.ac.uk

**Keywords:** human digital twin, human intention prediction, human motion generation, human–robot collaboration, artificial intelligence

## Abstract

In manufacturing settings where humans and machines collaborate, understanding and predicting human intention is crucial for enabling the seamless execution of tasks. This knowledge is the basis for creating an intelligent, symbiotic, and collaborative environment. However, current foundation models often fall short in directly anticipating complex tasks and producing contextually appropriate motion. This paper proposes a modular framework that investigates strategies for structuring task knowledge and engineering context-rich prompts to guide Vision–Language Models in understanding and predicting human intention in semi-structured environments. Our evaluation, conducted across three use cases of varying complexity, reveals a critical tradeoff between prediction accuracy and latency. We demonstrate that a Rolling Context Window strategy, which uses a history of frames and the previously predicted state, achieves a strong balance of performance and efficiency. This approach significantly outperforms single-image inputs and computationally expensive in-context learning methods. Furthermore, incorporating egocentric video views yields a substantial 10.7% performance increase in complex tasks. For short-term motion forecasting, we show that the accuracy of joint position estimates is enhanced by using historical pose, gaze data, and in-context examples.

## 1. Introduction

The paradigm of digital twins has found widespread application in optimizing performance across diverse industrial sectors, including manufacturing, aerospace, and energy [1,2]. However, conventional digital twins often prioritize technical system aspects, frequently overlooking the human element crucial for Industry 5.0. Human-Centric Digital Twins (HCDTs) aim to address this by integrating models of human behavior, cognition, and even emotion, enabling personalized and adaptive feedback to human operators [3]. A truly effective HCDT allows the predicted intention and motion to be utilized in several ways: for real-time monitoring of operator performance; for providing semantic assistance such as prompting the operator on the next step or flagging procedural errors; and for enabling a robotic assistant to proactively provide physical support. Such HCDTs promise to elevate safety, efficiency, and productivity in human–machine systems while concurrently enhancing user experience and satisfaction [4]. Traditional automation systems exhibit significant rigidity in High-Mix–Low-Volume (HMLV) industrial contexts, necessitating substantial human intervention for customization and adaptation. The advent of Machine Learning (ML), Artificial Intelligence (AI), and advanced automation technologies offers a pathway towards more flexible solutions, particularly for Small and Medium Enterprises (SMEs). These innovations can facilitate partial automation of labor-intensive tasks or enable collaborative human–robot workflows in which humans focus on complex reasoning and dexterous manipulation while robots manage repetitive actions [5,6]. Digital twins of these collaborative processes can further enhance skill transfer and enable remote oversight via Virtual Reality/Augmented Reality (VR/AR) [7,8]. Despite these technological strides, industrial robotics largely remains confined to preprogrammed caged systems, with collaborative applications still in their infancy. Recent breakthroughs in behavior cloning [9], diffusion policies [10], high-fidelity simulators [11,12], and the proliferation of Large Language Models (LLMs) [13,14] and Vision–Language Models (VLMs) [15] have expanded the horizons for semantic understanding, task planning, and perception in robotic systems [16,17].

Achieving robust spatial intelligence, such as forecasting detailed 3D human motion from visual inputs in partially structured settings, remains an important biomimetic challenge. This requires emulating the innate human ability to predict others’ future actions by synthesizing a rich stream of multimodal cues. For instance, when a person glances at an empty cup and then stands up, this indicates a likely intention to get a drink. Such social cognition relies on diverse sensory inputs, including body language, posture, tracking gaze direction, recalling recent actions to understand context, and applying prior knowledge about the task and environment. In order to create machines that can seamlessly and safely work alongside people, artificial systems must be able to emulate this predictive ability. Additionally, translating this understanding into accurate 3D motion prediction remains an open problem. This predictive capability is paramount for robots to transition from reactive assistance to smart collaboration.

To address this challenge, in this paper we propose a modular and scalable framework that utilizes Vision–Language Models (VLMs) with motion diffusion techniques to predict human intent and generate plausible future human motion in semi-structured environments. Instead of pursuing end-to-end training, this approach emphasizes the integration of existing pretrained perception and reasoning modules. This design philosophy not only makes the system more practical for SMEs with limited resources but also ensures adaptability, as overall performance can improve with advancements in individual modules. To validate this framework, three use case scenarios with varying levels of complexity are utilized, including scenarios from existing datasets such as HaVID [18] and EgoExo4D [19]. This provides a systematic evaluation of the framework’s ability to perform context-driven and intention-aware motion prediction. The remainder of this paper is structured as follows: Section 2 reviews related research; Section 3 details the proposed framework; Section 4 presents the validation approach and experimental results; finally, Section 6 discusses the implications of this work and outlines future directions.

## 2. Background and Related Work

The endeavor to create proactive robotic assistants capable of anticipating human needs and actions draws upon several interconnected research areas. This section reviews pertinent work in human intent prediction for Human–Robot Collaboration (HRC), AI-driven human motion generation, the role of LLMs in robotics, and the critical aspect of which datasets are available for these tasks.

### 2.1. Human Intent Prediction for Collaborative Environments

Anticipating human intent is pivotal to enhancing safety, security, ergonomics, and effective collaboration in Industry 5.0. Early work often focused on trajectory prediction in constrained scenarios [20]. More recent approaches have sought to infer higher-level goals. For instance, Huang et al. [21] proposed a hierarchical intention-tracking framework for assembly tasks, utilizing OpenPose and Kalman filtering to track wrist positions and infer both high-level task goals and low-level current actions. Similarly, Mangin et al. [22] developed hierarchical planners that infer human goals using partially observable Markov decision processes for procedural tasks. These methods underscore the importance of structured task understanding.

The integration of richer sensory data and more sophisticated AI models is an ongoing trend. Zhong et al. [23] introduced a framework for human–robot task handover that fuses a hierarchical human digital twin with deep domain adaptation while leveraging spatiotemporal graph convolutional networks. Ding et al. [24] proposed a dynamic scenario-enhanced network for predicting stochastic motions in customized assembly tasks, highlighting the need to handle variability. The use of egocentric data has also gained traction, with works like that of Mascaro et al. [25] developing intention-conditioned hierarchical architectures for long-term action anticipation based on the Ego4D dataset [26]. However, many existing methods focus on recognizing intent from past actions rather than on proactively forecasting future motion linked to that intent. They also often lack robust integration with real-time simulation for physical plausibility.

### 2.2. AI-Driven Virtual Human Motion Generation

Generating realistic and controllable human motion is a long-standing challenge in computer graphics and AI. The field has historically relied on motion capture (MoCap) datasets, which provide high-fidelity kinematic data for a wide range of human activities. A significant advancement was the creation of large-scale datasets such as HumanML3D that pair MoCap data with natural-language descriptions [27]. Pioneering works such as Adversarial Motion Priors (AMP) [28,29] trained Reinforcement Learning (RL) policies to perform tasks while using a discriminator to ensure that the resulting motions were realistic and stylistically similar to MoCap examples. This has been extended by models such as Adversarial Skill Embeddings (ASE) [30] and Conditional Adversarial Latent Models (CALM) [31], which focus on learning a low-dimensional latent space that can be sampled to direct a character’s behavior.

With the advent of kinematic diffusion models, the Human Motion Diffusion Model (MDM) [32] has demonstrated a remarkable ability to generate complex motions from text prompts alone. However, because they lack physical grounding, these purely kinematic approaches often produce physically implausible motions with artifacts such as foot-sliding, floating, and ground penetration. PhysDiff [33] introduced a novel physics-guided approach that incorporates a physics simulator directly into the diffusion process, thereby correcting the motion to adhere to physical constraints.

Recent work has focused on creating unified controllers that combine the strengths of generative models with the realism of physics-based simulation for more interactive and multimodal control. MaskedMimic [34] presented a unified framework that formulates physics-based character control as a versatile motion inpainting problem. It trains a single controller to synthesize physically plausible full-body motions from partial or “masked” inputs, which can include any combination of target joint positions, text commands, or object interactions. Another state-of-the-art approach is CLoSD [35], which closes the loop between motion planning and execution. It uses a real-time autoregressive Diffusion Planner (DiP) to generate kinematic motion plans on-the-fly, which are then executed by a robust RL-based tracking controller in a physics simulator. However, these MoCap-based methods often lack the rich scene-interaction context necessary for robustly forecasting actions in unstructured real-world environments.

### 2.3. The Role of LLMs in Intelligent Robots

The advent of LLMs has opened up new avenues for enhancing the semantic understanding and planning capabilities of robotic systems. LLMs can parse natural language instructions, reason about task goals, and even generate plans or code snippets for robotic execution [14,16]. For instance, SayCan [17] demonstrated how LLMs can propose high-level actions grounded by pretrained robotic skills. Singh et al. [16] developed ProgPrompt using programmatic LLM prompts to generate executable plans. Ha et al. [36] explored using LLMs to guide high-level planning for data collection, then distilling this into visuomotor policies. Further, efforts such as “Asking Before Action” by Chen et al. [37] empower LLM agents to proactively seek information. These works highlight LLMs’ potential in interpreting complex instructions and decomposing them into actionable steps. However, they are primarily designed to interpret high-level commands and map them to a robot’s own action space. They are not inherently structured to analyze a continuous stream of human motion and environmental context to proactively predict a human’s future intent and generate a corresponding physically plausible motion trajectory, which is the central focus of our work. The Magentic-One framework [38] showcases the potential of multi-agent systems driven by LLMs for complex task solving. However, grounding LLM outputs in the physical world, ensuring feasibility, and integrating them with continuous motion generation remain active research areas.

### 2.4. Datasets for Human Motion and Intent

The development of robust models for intention-based motion generation is intrinsically linked to the availability of suitable datasets. Motion capture (MoCap) datasets such as AMASS [39] and HumanML3D [27] offer precise 3D human kinematics. These datasets are invaluable for learning the fundamental dynamics and stylistic nuances of human movement. However, they are typically recorded in controlled laboratory settings, and often lack the rich object interactions and complex environmental context necessary for inferring high-level human intent. While some datasets, such as KIT Motion-Language [40], pair motion with textual descriptions, these descriptions usually label the overt action rather than the underlying intent or the broader task goal. Consequently, their utility for training models that predict intent from a wider range of cues is limited. Conversely, video-based datasets such as Ego4D [26], EgoExo4D [19], Assembly101 [41], and HaVID [18] provide a wealth of visual context, capturing humans performing tasks in more natural and often cluttered environments. Ego4D, with its extensive collection of egocentric video, offers a first-person perspective on daily activities. EgoExo4D complements this with synchronized exocentric views, providing a more holistic understanding of human actions and interactions within a scene. Assembly101 focuses specifically on procedural assembly tasks, offering structured sequences of actions. HaVID [18] contributes a dataset focused on human assembly with an emphasis on comprehensive knowledge understanding, including granular action annotations. These datasets are more conducive to understanding human–object interactions and inferring short-term goals or intentions from visual cues and task progression. However, extracting precise full-body 3D human motion comparable to MoCap quality from these “in-the-wild” videos remains a significant challenge, often relying on human pose estimation via methods such as Multi-HMR [42].

This reveals a critical gap in the lack of a unified framework capable of utilizing rich multimodal human observations such as visual data, pose dynamics, and gaze together with formal task knowledge. A bridge to connect the semantic long-term understanding derived from these inputs with a physically plausible forecast of future movements is currently lacking.

The present work directly addresses this gap by proposing a novel modular framework that creates a pipeline using the necessary context from these specific multimodal inputs for semantic intent prediction, ultimately providing context-aware motion synthesis. This approach provides a solution for proactive forecasting, a necessary capability for enabling symbiotic human–machine systems in complex environments.

## 3. Proposed Framework

A modular framework is proposed to enable intelligent HRC by predicting human intention and future pose. This methodology is designed to bridge the gap between short-term motion forecasting and long-term action planning, ensuring that predictions are both physically plausible and contextually appropriate.

Let the input at any given time *t* consist of a history of video frames $V={\left\{I_{k}\right\}}_{k=1}^{t}$, extracted human poses $P={\left\{P_{k}\right\}}_{k=1}^{t}$, and static high-level task knowledge $C_{task}$. The framework, represented by a function F, processes this context to provide three outputs: the predicted task state $S_{t}$, a forecast of future hand positions ${\hat{H}}_{t+\Delta t}=\left(\left(x_{l},y_{l}\right),\left(x_{r},y_{r}\right)\right)$ for a time horizon Δt, and a full-body 3D motion trajectory ${\hat{M}}_{t_{i}}=\left\{{\hat{x}}_{t_{i}+1},\ldots ,{\hat{x}}_{t_{i}+N_{\mathrm{pred}}}\right\}$.

**Figure 1:**
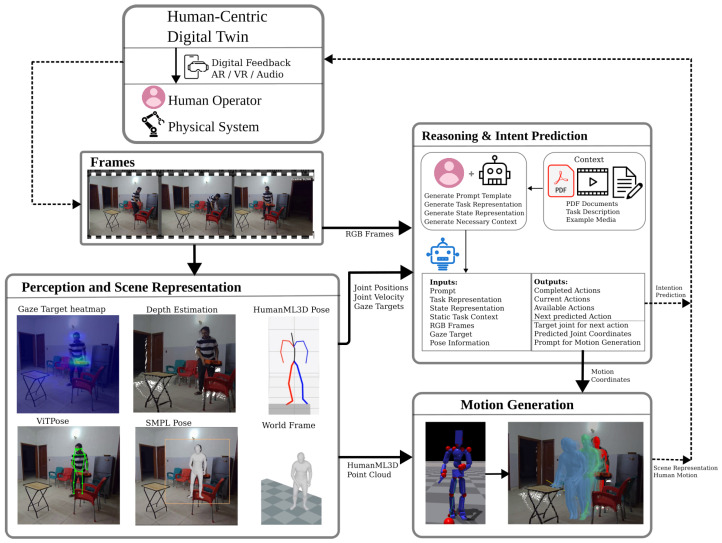
Conceptual overview of the proposed modular framework for Human Intention Prediction-based Motion Generation. Information flows from the physical system through the perception and reasoning modules to generate a final motion prediction.

As shown in Figure 1, the proposed framework has three primary modules: a Perception and Scene Representation Module that processes raw visual data; an AI-driven Reasoning and Prediction Module that infers task state and forecasts future actions; and a downstream Motion Generation Module that synthesizes full-body 3D motion. The specific functions of each of these modules are detailed in Section 3.1, Section 3.2 and Section 3.3. This modularity allows for the individual modules to be upgraded as technology advances. While the framework provides the necessary predictive outputs for a complete Human-Centric Digital Twin (HCDT), the implementation of feedback and physical assistance mechanisms is designated as future work.

### 3.1. Perception and Scene Representation

The framework adopts the Skinned Multi-Person Linear Model with Extensions (SMPL-X) [43], which provides a parametric model for body shape β, pose θ, and expressive hand and face parameters ψ. To obtain the pose parameters from monocular RGB video, we employ World-Grounded Human Motion Recovery via Gravity-View Coordinates (GVHMR) [42,44]. GVHMR reconstructs human pose in a global gravity-aligned coordinate system. For kinematic motion generation, the SMPL-X poses are subsequently converted into the representation utilized by HumanML3D [27]. This format captures frame-relative information such as root angular and linear velocities (${\dot{r}}_{a},{\dot{r}}_{x},{\dot{r}}_{z}$), root height ($r_{y}$), and local joint positions, rotations, and velocities ($j_{p},j_{r},j_{v}$), along with foot contact features (*f*). This relative encoding facilitates the generation of smooth and continuous motions.

**Figure 2:**
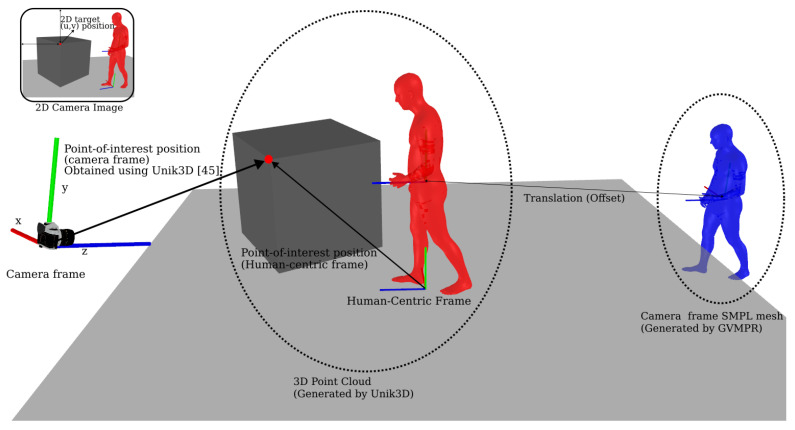
The 3D grounding process combines dense 3D from UniK3D [45] with the camera-to-world transform from GVHMR [44] to convert 2D pixel coordinates into 3D world coordinates.

Additionally, this module is required to convert the VLM’s 2D predictions into 3D world coordinates. To convert 2D predictions into 3D locations, we combine dense camera-frame 3D from UniK3D [45] with the rigid transforms produced by GVHMR. Figure 2 summarizes the process. UniK3D returns per-pixel 3D points $X_{c}\left(u,v\right)\in R^{3}$ for the input image. Additionally, GVHMR is used to find the transformation that maps the camera-frame to a gravity-aligned human-centric frame: $X_{h}=R_{c\to h}\;X_{c}+T_{c\to h}$. We render the camera frame SMPL mesh (from GVHMR) together with the scene point cloud (from UniK3D). An offset is estimated to co-locate the human in both modalities. We obtain the pelvis pixel $\left(u_{p},v_{p}\right)$ from ViTPose (used by GVHMR) and sample UniK3D at $\left(u_{p},v_{p}\right)$ to obtain the 3D coordinates of the pelvis position $p_{c}$. We compare $p_{c}$ with the pelvis position of the SMPL mesh in the camera frame and apply the resulting transformation to align the mesh with the point cloud. In our experiments, minor manual refinements of this alignment were applied as needed. Given a 2D point of interest (u,v), we compute

(1) $$X_{h}=R_{c\to h}\;X_{c}\left(u,v\right)+T_{c\to h}.$$

The resulting 3D target is passed to the motion module as the goal position for a selected joint (e.g., wrist or pelvis), as further described in Section 3.3.

For specialized applications requiring different or higher-precision grounding, this module can be extended to incorporate other engineered approaches, such as processing fiducial markers.

This module also incorporates human gaze target analysis, which provides a cue for immediate intent. A change in the operator’s gaze target is often a useful indicator of a change in intention, and the location of the gaze target frequently correlates with the area or object that the operator intends to interact with next. Techniques such as Gaze-LLE [46] can be employed for gaze target tracking to further enrich the context provided to the reasoning and intent prediction module.

### 3.2. AI-Driven Reasoning and Intent Prediction

This module serves as the cognitive core of the framework. The integration of VLMs for high-level reasoning about tasks and intent requires appropriate data structures. Task dependencies are modeled using formalisms such as Directed Acyclic Graphs (DAGs), which define the valid sequence of actions and states. Alternative formalisms, such as Planning Domain Definition Language (PDDL) [16], Knowledge Graphs (KGs), or Behavior Trees (BTs) [17,47], can also be integrated. For any use case, a common operational flow is followed. Initially, task-specific knowledge such as assembly instructions, procedural steps, or an example video is provided to the system. This involves a collaborative effort in which an AI model generates an initial draft of a task representation (i.e., an action graph and state schema), which is then refined by human experts. This task representation forms a critical part of the context, encompassing the overall goal, a mechanism for state tracking, decomposition into smaller actions, and their respective dependencies.

**Figure 3:**
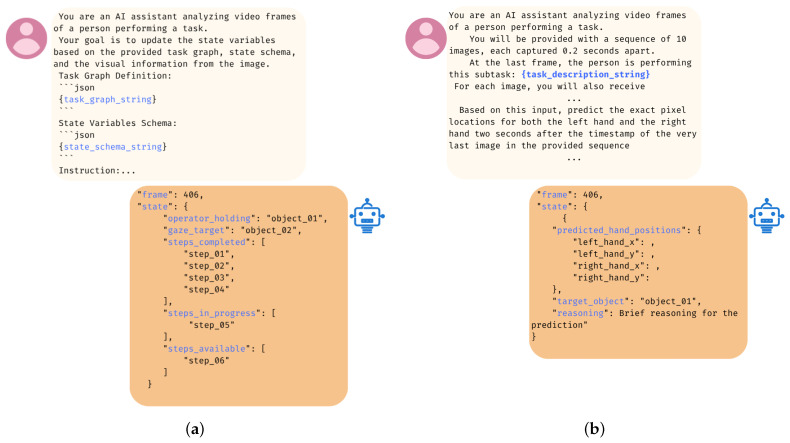
The two-phase pipeline of the Reasoning and Intent Prediction Module. (**a**) In the first phase, the VLM uses the task graph and visual information to update the overall task state; (**b**) in the second phase, it uses this state along with a sequence of recent frames and additional context to predict the operator’s future hand positions.

The prediction process follows a two-phase approach. The VLM analyzes video frames and appropriate context from the perception module along with the task graph in order to first output an updated Task State in a structured JSON format. Here, the system identifies the completed steps, in-progress steps, available steps, and immediate next step. In the second phase, the VLM uses this updated task context and multimodal data from the perception module to forecast the exact pixel coordinates (*x*, *y*) for both hands of the operator at a future time horizon. This two-phase pipeline is illustrated in Figure 3. This two-phase approach is crucial because it decouples long-horizon semantic reasoning about the overall task from short-horizon spatiotemporal forecasting of immediate motion, allowing each phase to be optimized with the most relevant context. In this module, various prompting strategies are investigated to optimize the VLM’s predictive accuracy, which are discussed in Section 5.

### 3.3. Human Motion Generation

As a final, downstream step, the high-level predictions from the Reasoning and Intent Prediction Module are used to drive the Motion Generation Module. This module is crucial for visualizing the predicted intent as a full-body 3D motion. The core of this module adapts the CLoSD framework [35], which builds upon the Human Motion Diffusion Model (MDM) [32]. The CLoSD framework utilizes a forward diffusion process $q\left(x_{t}|x_{t-1}\right)=N\left(x_{t};\sqrt{\alpha_{t}}x_{t-1},\left(1-\alpha_{t}\right)I\right)$ that gradually adds noise to a motion sequence, while the reverse process $p_{\theta }\left(x_{t-1}|x_{t}\right)=N\left(x_{t-1};\mu_{\theta }\left(x_{t},t\right),\sigma_{t}^{2}I\right)$ learns to denoise it by minimizing an L2 loss $L_{\mathrm{simple}}=E_{x_{0},t}[∥x_{0}-{\hat{x}}_{0}{∥}_{2}^{2}]$. The model receives the previous 40 frames of motion data in HumanML3D format and synthesizes the future motion sequences for the next two seconds (60 frames). This is additionally conditioned on a textual prompt and a target joint (e.g., right_wrist, pelvis) and its target 3D location. Hence, the Human Motion Model synthesizes a physically plausible and semantically guided future motion sequence. This motion sequence is subsequently visualized in a virtual representation of the real environment provided by a 3D point cloud created by the perception module’s depth estimation model.

## 4. Framework Validation and Use Cases

This section validates the proposed framework by applying it to three diverse use cases. Each scenario is designed to test the framework’s predictive capabilities in a different context of varying difficulty.

### 4.1. Use Case 1: Chair Stacking Scenario

**Figure 4:**
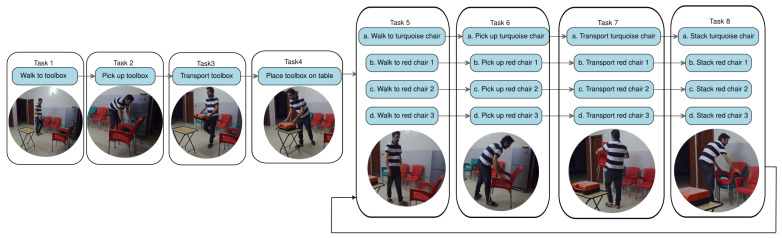
Use Case 1: Stacking task in a domestic setting.

This use case introduces a predictable task to demonstrate the framework’s core capabilities: a person moving a toolbox from a chair and then stacking several chairs in a domestic environment, as shown in Figure 4.

The initial phase involves human–AI collaboration to define the task context. An AI model analyzes a sample video of the task or textual instructions to propose an initial “Task Context Description”. This description includes the overall goal (e.g., “stack all chairs in the designated area”), a state representation (e.g., operator holding status, ordered list of stacked chairs), a decomposition of actions (e.g., “walk to turquoise chair”, “pick up red chair”, “transport turquoise chair”), and their dependencies. This draft is then reviewed and refined by a human. The LLM’s output is a JSON object representing the updated state variables based on the task graph, including gaze_target, operator_holding, num_chairs_stacked, steps_completed, and steps_available.

**Figure 5:**
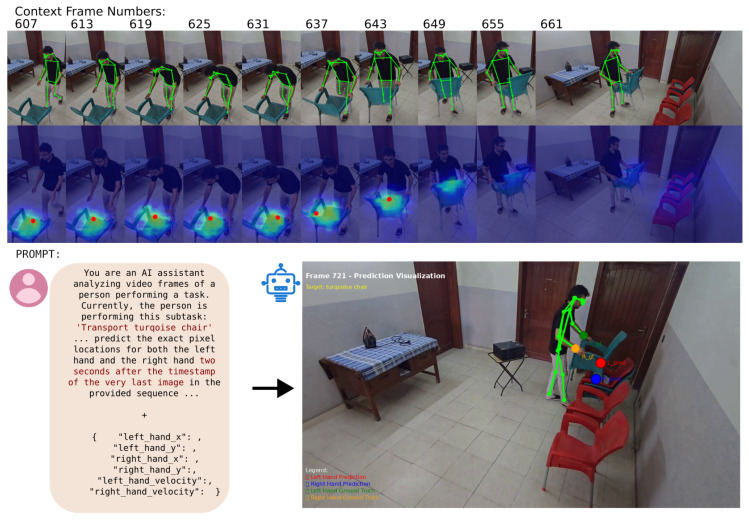
Hand position prediction for Stacking scenario.

In the first phase, the overall task state is predicted by the framework. When the overall task state is understood, the framework can proceed to a more granular short-term motion prediction task, as illustrated in Figure 5. For this, the VLM is provided with a rich multimodal context. This includes a sequence of the ten most recent image frames, captured at intervals of 0.2 s. Each frame is augmented with vital information: a heatmap indicating the operator’s gaze target, an overlay of the estimated human pose, and the precise normalized pixel coordinates and velocities of the operator’s hands. The model is also provided with the current sub-task description in text form (e.g., “transport turquoise chair”). The VLM’s objective is to analyze this temporal sequence, then, informed by the task context, to predict the exact pixel locations of the left and right hands two seconds after the final frame in the sequence. This approach moves beyond simple kinematic extrapolation, compelling the model to make a physically and semantically plausible forecast based on an understanding of the human’s immediate goal.

**Figure 6:**
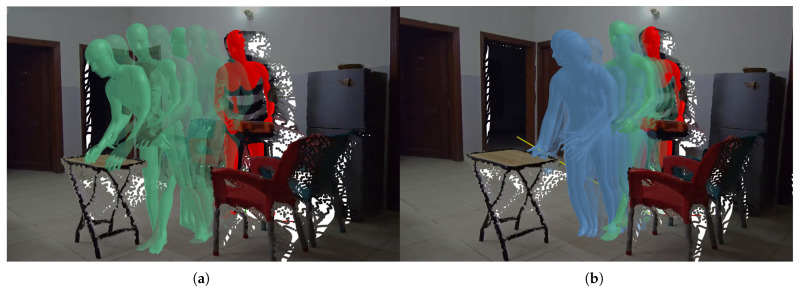
Visualizations for Task 4 within the Stacking use case. (**a**) Starting position (in red) and ground truth of the human’s motion (in green) as they place the toolbox on the table; (**b**) generated motion (in blue), with the yellow line representing the target vector for the position of the left wrist.

Depending on the approach, the *x*, *y* pixel coordinates or the target object can be processed to find the world-frame coordinates of the target joint, as described in Section 3.1. This is further employed in the motion generation module. Figure 6 illustrates the motion generation results for the chair-stacking use case while performing Task 4: “place toolbox on chair”.

**Figure 7:**
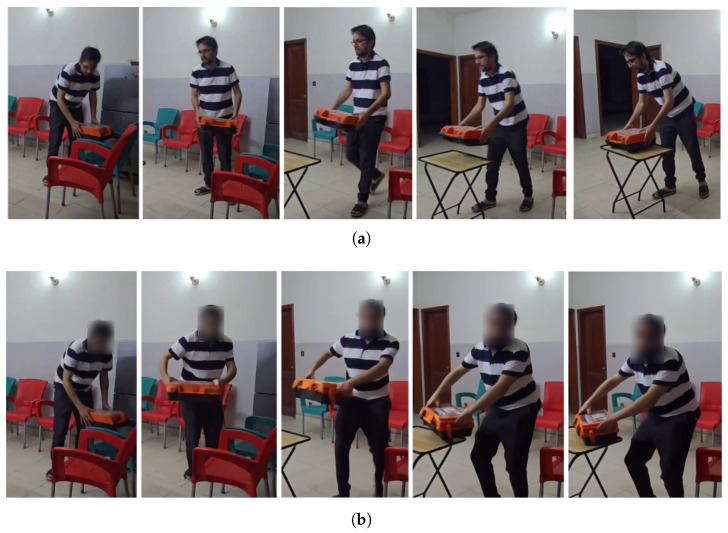
Comparison of actual and AI-generated motion frames for placing a toolbox: (**a**) actual motion frames and (**b**) AI-generated motion frames.

The modularity of the framework also allows for flexible integration of alternative motion visualization methods. One such alternative is the use of AI driven image-to-video generation tools. In this approach, the reasoning and intent prediction module of the framework generates a text prompt describing the predicted intent. The prompt and the starting frame of the sequence are passed to a video generation model. This method is not suitable for real-time prediction due to significant computational latency. Additionally, it can sometimes lead to physically implausible hallucinations. However, it serves as a powerful tool for visualization. Figure 7 illustrates a comparison between actual motion frames and those synthesized by the Wan2.1 framework [48] using this technique.

### 4.2. Use Case 2: Assembly Task

**Figure 8:**
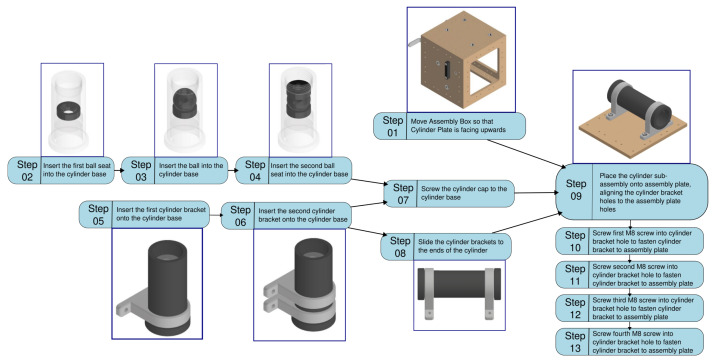
Directed Action Graph (DAG) for Assembly Task based on the HAViD dataset.

The second use case leverages the Human Assembly Video Dataset (HaVID) [18] to evaluate the framework’s performance on fine-grained industrial assembly tasks. HaVID’s detailed annotations of actions provide a rich ground truth for assessing intent prediction. This use case demonstrates the framework’s applicability to tasks with clear SOPs, common in manufacturing environments [49]. PDF assembly instructions and exploded assembly drawings for a task such as HaVID’s “Cylinder Assembly” are first used with AI assistance to generate a comprehensive action graph (DAG) and a suitable state representation (Figure 8). Given the potential for variations in assembly sequences, multiple valid paths through the DAG are considered.

**Figure 9:**
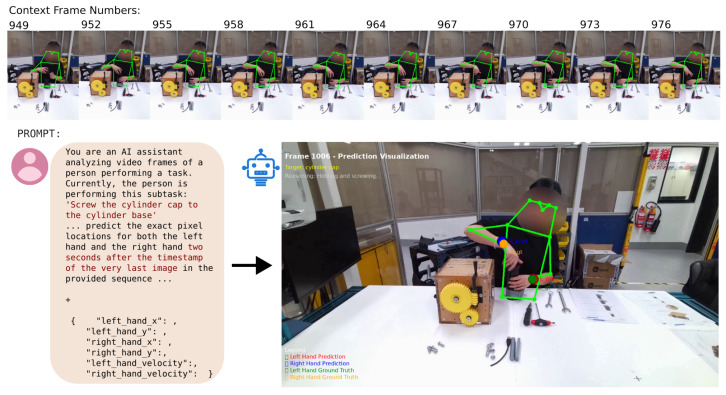
Hand position prediction for Assembly scenario.

Ground truth states for selected HaVID video segments are then generated by translating HaVID’s temporal annotations into the state representation format in a process facilitated by AI with human oversight. These ground truth states serve for in-context learning examples as well as for evaluation. As detailed in the previous use case, the system first predicts the system state. This is followed by a fine-grained prediction of hand positions to forecast the operator’s immediate movements, applying the same multimodal analysis approach (Figure 9).

**Figure 10:**
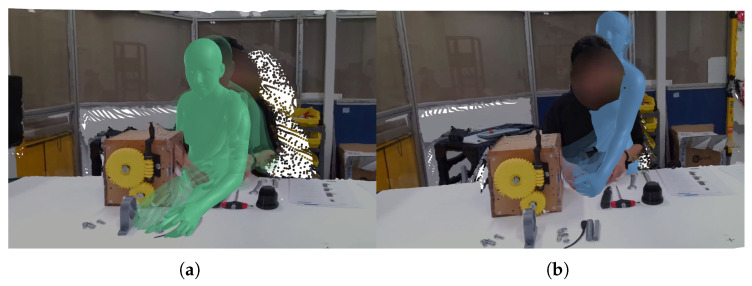
Visualizations within the HAVID Assembly use case. (**a**) Ground truth of the human’s motion over the previous 1 s (in green) and (**b**) generated motion (in blue), indicating the expected motion of the human’s hand.

The results of this intent-aware forecasting are then used to generate plausible future motion, as visualized in Figure 10.

### 4.3. Use Case 3: Cooking Scenario

To test the framework against long-horizon and complex tasks, the third use case utilizes the EgoExo4D dataset [19]. This dataset is particularly suitable for this research because it includes synchronized egocentric and exocentric video streams along with rich temporal annotations, including task and keystep-level labels. A challenging noodle cooking scenario was selected for which multiple recordings from the same kitchen location were available, providing ideal in-context learning examples.

**Figure 11:**
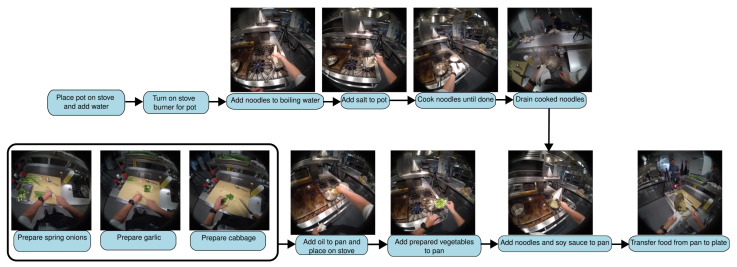
Sample action graph for Cooking scenario.

The cooking task (Figure 11) introduces distinct challenges that are not as prevalent in the other scenarios. It involves a much larger time horizon, and the state of the task is often ambiguous and not easily discernible from visual cues alone. For instance, by observing a single frame or even a short sequence of an idle cook, it is difficult to determine if the vegetables are fully chopped or if the noodles have finished boiling; furthermore, the scenario includes significant periods of inactivity or “waiting” during which the operator’s next action is not imminent. While the overall task has a clear structure, the cook does not need to move from completing one sub-task to immediately carrying out the next, making the precise timing of future actions inherently unpredictable.

**Figure 12:**
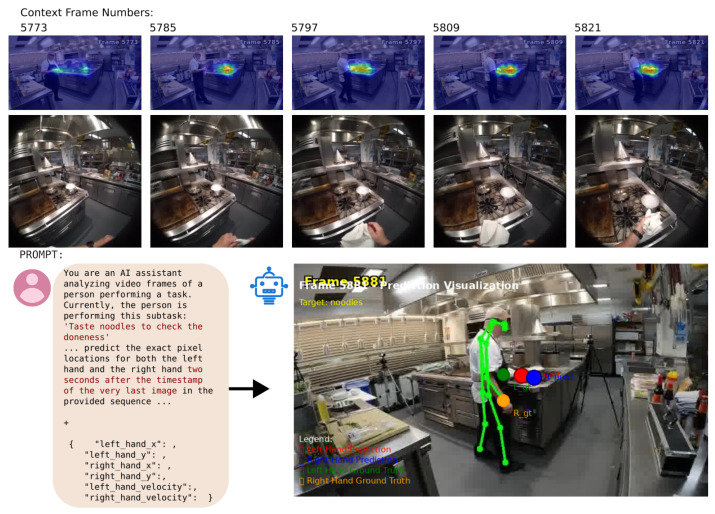
Hand position prediction for Cooking scenario.

Despite these complexities, the framework follows the established two-phase prediction process, with the AI agent first predicting the current action or task state, then using this to inform a fine-grained hand position prediction. A key distinction in this use case is the augmentation of the VLM’s context with first-person egocentric frames, which are provided to the model along with the primary exocentric view and gaze data, as shown in Figure 12. For brevity, only half the context frames are shown in the figure. The RGB frames provided as context are also omitted.

## 5. Framework Performance Evaluation

Evaluating the performance of the proposed framework requires a multi-faceted approach, as no single established benchmark currently exists for the end-to-end task of context-aware human intention and motion prediction. Our work aims to lay the groundwork for such a benchmark; therefore, we define a set of specialized metrics to assess both the high-level semantic understanding (Phase 1) and low-level motion prediction accuracy (Phase 2), and report results across diverse use cases.

To evaluate the frame-wise prediction of the task state, we use a weighted F1-score, which is well-suited for this multi-label classification problem [50]:

(2) $$F1=2\cdot \frac{P\cdot R}{P+R}=\frac{2TP}{2TP+FP+FN}$$

where TP, FP, and FN are the counts of true positives, false positives, and false negatives, respectively, and *P* and *R* are the precision and recall, respectively. The overall Task State Accuracy (TSA) is calculated as a weighted sum of the F1-scores for the classification of completed steps, in-progress steps, available steps, and the immediate next step, as follows:

(3) $$\mathrm{TSA}\;\mathrm{Score}=(w_{c}F1_{c}+w_{p}F1_{p}+w_{a}F1_{a}+w_{n}F1_{n})$$

where $F1_{c},F1_{p},F1_{a}$, and $F1_{n}$ are the F1-scores for the completed, in-progress, available, and immediate next steps, respectively, with weights $w_{c},w_{p},w_{a}$, and $w_{n}$.

Table 1 presents performance figures for the Task State Accuracy. A central observation is the tradeoff between accuracy, latency, and cost. A baseline strategy of providing only a single image to the Vision–Language Model (VLM) consistently underperforms due to the lack of historical context or “memory”. For a balance of performance and latency, a Rolling Context Window (RCW) strategy was employed using ten frames spaced one second apart, augmented with the predicted state from the initial frame. However, this approach is prone to consistency bias; if the model erroneously determines that a step has been completed, it often fails to revise this belief, causing subsequent predictions to suffer. When this strategy is tested with the ground-truth state provided as context, performance is significantly higher and becomes less dependent on model size, with smaller models also performing well. This approach can achieve near real-time predictions (within 2–3 s) when using the low-latency Gemini 2.5 Flash-Lite model, whereas Gemma and the standard Gemini 2.5 Flash models average closer to 10 s. The best accuracy is achieved using in-context learning, where a similar full example video and corresponding evolution of its ground-truth state are provided in the prompt. While effective, the associated latency makes this approach impractical for real-time applications with current technology.

The inclusion of additional visual cues yielded mixed results. A significant improvement was observed by incorporating egocentric first-person views in the EgoExo4D-based Cooking scenario, where performance increased from 0.568 to 0.629, a 10.7% improvement. In contrast, adding gaze target heatmaps, when averaged over all experiments, led to only an inconsistent and marginal 3% performance increase, from 0.667 to 0.688. We theorize that the egocentric perspective provides a more reliable signal of operator attention than gaze target heatmaps. In addition, this suggests that while gaze data are valuable, their primary utility may be in predicting short-term hand motion, as discussed later, rather than long-horizon task state analysis.

**Figure 13:**
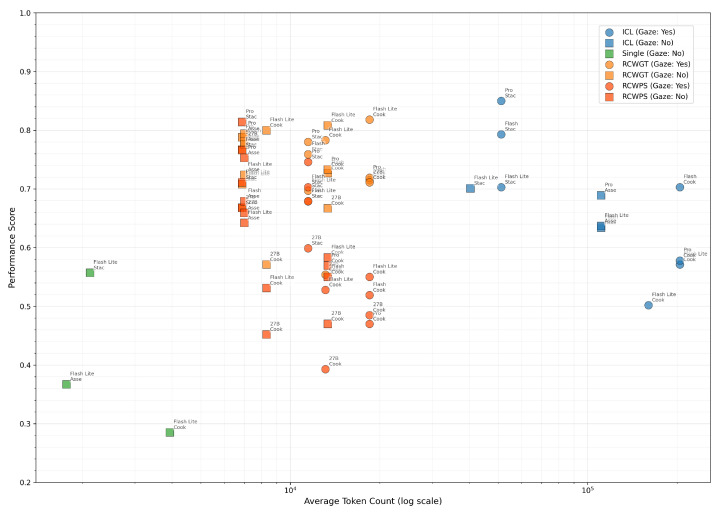
Performance score versus the average number of tokens per generation.

An effective strategy for LLMs should achieve high performance with minimal computational cost. Figure 13 illustrates the tradeoff between performance and the average number of tokens generated, which is a proxy for computational expense.

While providing more context can enrich a model’s understanding, this approach is not without drawbacks. In addition to the obvious increases in latency and cost, simply allocating more test-time compute by expanding the context can be actively detrimental to performance. Recent work has highlighted the phenomenon of inverse scaling in test-time compute, where model performance paradoxically decreases as they are provided with more computational resources to generate a response [51]. This suggests that models can “overthink” or become lost in an unnecessarily large context, leading to performance degradation. The ICL strategy, with its massive token count, runs a higher risk of encountering this issue. By being more selective with the provided context, RCW methods not only reduce latency and costs but also mitigate the risk of inverse scaling, demonstrating a more robust and efficient approach.

**Figure 14:**
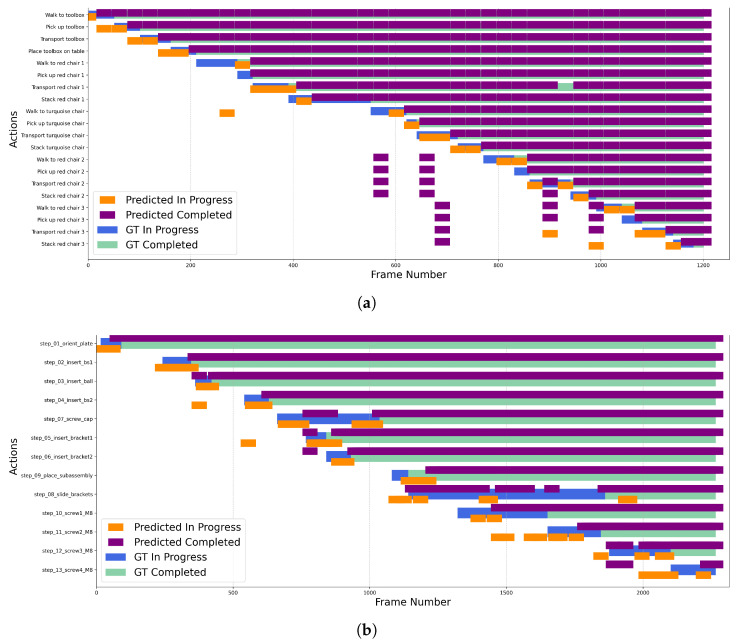

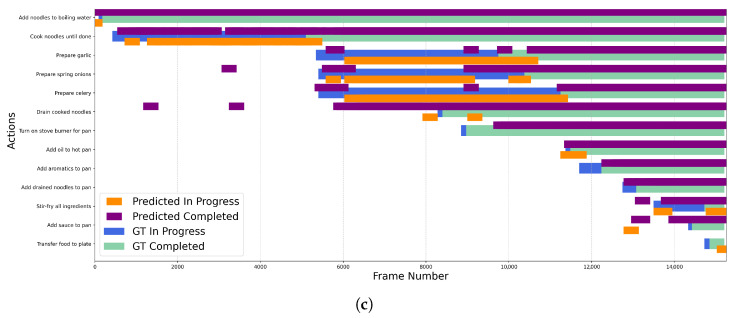
Visualization of model performance across different use cases: (**a**) Stacking, (**b**) Assembly, and (**c**) Cooking.

The temporal aspect of the predictions is critical. We plotted the timeliness of the model’s determination that a step was completed for each use case. The results show good agreement for the Stacking and HaVID scenarios; however, the prediction of subtask completion times for the Cooking scenario is less accurate, which is attributed to the ambiguous nature and prolonged waiting periods inherent to that task. The visualization for the best-performing models for each use case is shown in Figure 14.

For the second phase, consisting of predicting future hand positions, the Normalized Mean Position Error (NMPE) is used. This metric calculates the average L2 norm (Euclidean distance) of the pixel error between the predicted and ground-truth hand coordinates, normalized from 0 to 1000:

(4) $$\mathrm{NMPE}=\frac{1}{N}\sum_{i=1}^{N}\left(\frac{{\sqrt{{\left(x_{p,l}-x_{g,l}\right)}^{2}+{\left(y_{p,l}-y_{g,l}\right)}^{2}}}_{i}+{\sqrt{{\left(x_{p,r}-x_{g,r}\right)}^{2}+{\left(y_{p,r}-y_{g,r}\right)}^{2}}}_{i}}{2}\right)$$

where the subscripts p,g,l, and *r* respectively denote predicted, ground-truth, left hand, and right hand for each prediction instance *i* out of *N*.

**Figure 15:**
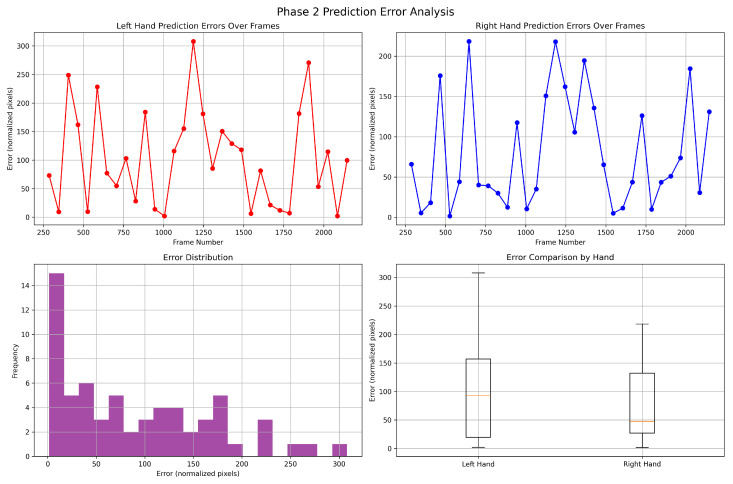
Sample Error Analysis for hand position prediction in Assembly scenario.

While NMPE measures accuracy, it does not capture the exploratory nature of a model’s predictions. A model that simply predicts minimal movement from the last known position might achieve a low NMPE but fail to anticipate significant goal-directed actions. To quantify this, a Prediction Diversity metric is introduced. This metric measures how far a prediction deviates from the recent trajectory, rewarding predictions that are not mere extrapolations. It is calculated as the average of the Euclidean distances from the predicted point to both the average position and the final position of the hand in the input sequence, normalized by the standard deviation of the input positions:

(5) $$\mathrm{Diversity}=\frac{D\left(p_{\mathrm{pred}},{\bar{p}}_{\mathrm{in}}\right)+D\left(p_{\mathrm{pred}},p_{\mathrm{last}}\right)}{2\sqrt{\sigma {\left(p_{\mathrm{in},x}\right)}^{2}+\sigma {\left(p_{\mathrm{in},y}\right)}^{2}}}$$

where $p_{\mathrm{pred}}$ is the predicted position, ${\bar{p}}_{\mathrm{in}}$ is the average position of the input trajectory, and $p_{\mathrm{last}}$ is the last position in the input trajectory. Figure 15 shows the detailed error analysis for a sample experiment using the Assembly scenario.

The performance of our two-phase pipeline is detailed in Table 2, which presents a breakdown of the Normalized Mean Position Error (NMPE) and Prediction Diversity across various use cases. The NMPE serves as our primary metric for motion prediction accuracy, while the Prediction Diversity quantifies the variability of the generated motion forecasts. A higher diversity score indicates the model’s capacity to predict significant goal-directed actions rather than simple extrapolations. Our findings show a consistent tradeoff between these two metrics, with higher prediction diversity often coming at the cost of increased position error. Additionally, we include the results of an ablation experiment using the Assembly scenario. In this “No Context” experiment, we performed hand position prediction without the task context from Phase 1. This resulted in a substantial degradation in performance, with the NMPE increasing to 202.35. This outcome underscores the necessity of our two-phase approach, which decouples long-horizon semantic reasoning from short-horizon spatiotemporal forecasting. Furthermore, our results consistently showed that few-shot predictions with one or two in-context examples provided significant accuracy improvements over a zero-shot approach.

## 6. Discussion and Future Work

The proposed modular framework advances the development of proactive robotic assistants by integrating state-of-the-art Vision–Language Models (VLMs) for high-level intent prediction with advanced models for perception and motion synthesis. This approach addresses key limitations in prior work. Unlike action recognition models, which are confined to limited classes [52,53] or context-agnostic pose forecasting methods [54,55], our framework leverages the generalized reasoning capabilities of VLMs to interpret multimodal context. By engineering this context through structured task graphs and rich perceptual data, the system can infer human intent in a way that is more aligned with the complexities of real-world tasks.

Prior work in social and assistive robotics often incorporates human intention within a narrow scope, such as to assess a user’s engagement level [56] or emotional state [57] and respond appropriately. Such unstructured interactions do not involve completing a specific industrial task or adhering to formal Standard Operating Procedures (SOPs). In contrast, our framework is designed to predict task-based intentions within structured goal-oriented workflows.

Despite its promise, the proposed framework has several limitations that can guide future research. The framework’s current reliance on predefined task graphs, even when AI-assisted, restricts its applicability in completely unstructured or novel scenarios where a task plan is not available beforehand. Furthermore, the motion generation pipeline is not object-aware; while it utilizes the CLoSD model [35] for physically plausible motion synthesis, the generated motions do not explicitly account for geometric interactions with specific objects in the environment. We address this limitation pragmatically by decoupling scene understanding from motion synthesis. The VLM, which is object-aware through its analysis of visual input, predicts a target 3D coordinate for a joint (e.g., placing a hand on a specific object). This 3D point then serves as a goal for the object-agnostic motion generation module. While this approach functions as an effective workaround, a fully integrated object-aware motion model would be superior. Future work will explore integrating emerging models for physics-based human–object interaction within our modular framework. However, these methods often rely on datasets with a limited variety of objects and interactions [58,59,60]. Finally, the use cases are centered on a single human operator, whereas many industrial environments involve complex multi-human collaborations, which introduces additional challenges such as occlusion and the need to interpret social dynamics.

To address these limitations and expand the framework’s capabilities, several avenues for future work are identified. A limitation of the current implementation is its reliance on a fixed-interval prediction cycle where motion is forecast at regular time steps regardless of the task’s dynamic context. Instead, an event-driven prediction framework can be explored where inference is triggered by salient cues, such as a sudden shift in gaze or the completion of a task step.

To improve generalization, methods for dynamically generating and adapting task graphs from observation may be explored, enabling the system to reason about unfamiliar workflows. Techniques inspired by multi-agent systems research [38] could offer novel ways to coordinate the flow of information and aid decision-making. However, this approach is expected to add more latency to the system. Prompting strategies might be explored to encourage the VLM to recognize uncertainty and proactively seek clarification when its confidence is low or when critical information is missing, drawing inspiration from approaches such as [37]. Incorporating mechanisms for learning from human feedback or corrections during interaction [61] could allow the system to adapt and respond to task requirements and individual preferences. Further research could also explore the integration of additional context modalities such as audio, physiological signals, or data from IoT sensors to create a more holistic understanding of the operational environment.

Another promising future direction is to use our framework’s proactive capabilities to enhance reactive impedance controllers for human-guided robots. By generating an anticipatory signal of where the user intends to move, an impedance controller can optimize its response in advance rather than reacting solely to force. This would lead to a more symbiotic physical interaction with reduced human effort and improved task performance [62]. In addition to predicting human intent for proactive assistance, the accurate forecasting of hand positions would also provide a critical safety layer for HRC. Anticipating human movement could enable robots to adjust their trajectories or speeds to avoid collisions, contributing to enhanced safety during human–robot collaboration. This capability is crucial for the adoption of heavy-payload robots in collaborative manufacturing, where safety concerns have historically been a major hurdle [63].

HCDTs powered by such human-centric situational awareness can serve as a virtual mentor for training. In addition, they can predict and flag ergonomically hazardous movements to prevent workplace injuries, and provide proactive robotic support by anticipating the need for assistance or carrying out tasks in parallel. By predicting human intent within a task context, our model provides a necessary prerequisite for systems in which a robot can intelligently assist a human worker without being explicit commanded. This capability is a key component of Industry 5.0, enabling resilient and adaptable manufacturing; furthermore, this paradigm moves us closer to prompt-based manufacturing, where high-level human intent expressed through natural language or gesture can be seamlessly translated into a machine’s adaptable physical actions. This approach is essential for enabling High-Mix–Low-Volume (HMLV) production and a more human-centric industrial automation. Critically, the transition from research prototype to a practical industrial tool depends on rigorous real-world validation. Future work must involve deployment in real-time lab-based HRC scenarios, where performance is assessed not only by prediction accuracy but also by HRC-centric metrics such as task efficiency, human idle time, and operator trust. Ultimately, this could enable a transition of robots from passive collaborative robots to intelligent coworkers.

## 7. Conclusions

This paper presents a modular framework for context-aware human intent prediction and subsequent 3D motion generation, with the aim of enabling proactive robotic assistance. By integrating pretrained Vision Language Models with state-of-the-art perception and generation modules, the proposed approach provides a scalable solution that bridges high-level semantic reasoning with physically grounded motion synthesis.

Our evaluation across three use cases of varying complexity revealed critical tradeoffs between predictive accuracy, latency, and diversity. In-Context Learning (ICL) demonstrates notable limitations that counteract its potential benefits. The requirement for a large context token count results in significant computational latency, in some cases leading to a paradoxical degradation of predictive accuracy that renders the approach unsuitable for real-time applications. We identified the Rolling Context Window (RCW) strategy as a more viable approach, offering a strong balance of performance and efficiency; however, this method is susceptible to consistency bias, as an initial error in state prediction can propagate and degrade subsequent performance. A key finding was that augmenting context with egocentric video views yielded a substantial 10.7% performance increase in complex tasks.

Furthermore, we observed a tradeoff between accuracy and prediction diversity when forecasting short-term motion. More powerful models tended to generate predictions with higher diversity, attempting to forecast significant goal-directed actions rather than simple extrapolations. However, this increased diversity often came at the cost of higher position error. Additionally, predictive accuracy showed consistent improvement when providing one or two in-context examples (few-shot) compared to a zero-shot approach.

Through continued development and integration, the framework presented in this work can significantly advance the ability of robots to understand, anticipate, and effectively collaborate with humans in complex dynamic settings.

## Figures and Tables

**Table 1:** Aggregated performance scores and rankings for Task State Accuracy.

		Task-Specific Score			Performance	
Exp. Group	Model	Stack	Assembly	Cooking	Overall	Rank
Single Image	Gemini 2.5 Flash Lite	0.557	0.367	0.285	0.403	
RCW with Ground Truth	Gemini 2.5 Pro	0.784	0.794	0.726	0.768	1
	Gemini 2.5 Flash	0.763	0.781	0.721	0.755	2
	Gemini 2.5 Flash Lite	0.703	0.724	0.813	0.747	3
	Gemma-27B	0.674	0.776	0.689	0.713	4
In-Context Learning	Gemini 2.5 Flash	0.793	0.634	0.703	0.710	1
	Gemini 2.5 Pro	0.850	0.689	0.578	0.706	2
	Gemini 2.5 Flash Lite	0.703	0.637	0.571	0.637	3
RCW with Predicted State	Gemini 2.5 Pro	0.780	0.753	0.520	0.684	1
	Gemini 2.5 Flash	0.735	0.679	0.535	0.650	2
	Gemini 2.5 Flash Lite	0.695	0.642	0.567	0.635	3
	Gemma-27B	0.634	0.659	0.478	0.590	4

**Table 2:** Normalized Mean Position Error (NMPE) and Prediction Diversity by use case.

Use Case	Model	Gaze Data	Examples	NMPE	Diversity
Stack	Gemini 2.5 Flash Lite	Yes	2-shot	109.46	2.02
	Gemini 2.5 Flash	Yes	2-shot	111.71	3.48
	Gemma 3 27B	Yes	0-shot	114.97	2.33
	Gemini 2.5 Flash Lite	No	2-shot	120.58	2.08
	Gemini 2.5 Flash Lite	Yes	0-shot	121.74	1.42
	Gemini 2.5 Pro	Yes	2-shot	152.68	5.68
	Gemini 2.5 Pro	Yes	1-shot	181.76	6.14
Assembly	Gemma 3 27B	N/A	1-shot	72.19	0.81
	Gemini 2.5 Flash Lite	N/A	2-shot	74.70	1.20
	Gemini 2.5 Flash Lite	N/A	0-shot	76.04	1.27
	Gemini 2.5 Flash	N/A	2-shot	81.85	3.67
	Gemini 2.5 Pro	N/A	1-shot	139.40	12.49
	Gemini 2.5 Pro (No Context)	N/A	0-shot	202.35	23.05
Cooking	Gemini 2.5 Flash Lite	Yes	0-shot	70.16	1.04
	Gemini 2.5 Flash Lite	Yes	1-shot	70.88	1.19
	Gemini 2.5 Flash Lite	No	1-shot	74.49	1.31
	Gemini 2.5 Flash	Yes	2-shot	78.36	2.69
	Gemini 2.5 Pro	Yes	1-shot	89.34	3.37

## Data Availability

Additional materials, videos, and the open-source implementation are available at the project website: https://usmanasad88.github.io/hcdt/ (accessed on 11 September 2025).

## Abbreviations

The following abbreviations are used in this manuscript:

AI	Artificial Intelligence
AR	Augmented Reality
BT	Behavior Tree
DAG	Directed Acyclic Graph
HCDT	Human-Centric Digital Twin
HMLV	High-Mix–Low-Volume
HRC	Human–Robot Collaboration
ICL	In-Context Learning
KG	Knowledge Graph
LLM	Large Language Model
ML	Machine Learning
MoCap	Motion Capture
NMPE	Normalized Mean Position Error
PDDL	Planning Domain Definition Language
POI	Point of Interest
RCW	Rolling Context Window
SME	Small and Medium Enterprises
SMPL-X	Skinned Multi-Person Linear Model with Extensions
TSA	Task State Accuracy
VLM	Vision–Language Model
VR	Virtual Reality
